# Effect of Pumpkin Seed Oil on Hair Growth in Men with Androgenetic Alopecia: A Randomized, Double-Blind, Placebo-Controlled Trial

**DOI:** 10.1155/2014/549721

**Published:** 2014-04-23

**Authors:** Young Hye Cho, Sang Yeoup Lee, Dong Wook Jeong, Eun Jung Choi, Yun Jin Kim, Jeong Gyu Lee, Yu Hyeon Yi, Hyeong Soo Cha

**Affiliations:** ^1^Family Medicine Clinic and Research Institute of Convergence of Biomedical Science and Technology, Pusan National University Yangsan Hospital, Beomeo-ri, Mulgeum-eup, Yangsan 770-626, Gyeongsangnam-do, Republic of Korea; ^2^Medical Education Unit, Pusan National University School of Medicine, Yangsan 626-870, Republic of Korea; ^3^Department of Family Medicine, Pusan National University Hospital, Busan 602-739, Republic of Korea; ^4^Centum Family Clinic, Busan 612-020, Republic of Korea

## Abstract

Pumpkin seed oil (PSO) has been shown to block the action of 5-alpha reductase and to have antiandrogenic effects on rats. This randomized, placebo-controlled, double-blind study was designed to investigate the efficacy and tolerability of PSO for treatment of hair growth in male patients with mild to moderate androgenetic alopecia (AGA). 76 male patients with AGA received 400 mg of PSO per day or a placebo for 24 weeks. Change over time in scalp hair growth was evaluated by four outcomes: assessment of standardized clinical photographs by a blinded investigator; patient self-assessment scores; scalp hair thickness; and scalp hair counts. Reports of adverse events were collected throughout the study. After 24 weeks of treatment, self-rated improvement score and self-rated satisfaction scores in the PSO-treated group were higher than in the placebo group (*P* = 0.013, 0.003). The PSO-treated group had more hair after treatment than at baseline, compared to the placebo group (*P* < 0.001). Mean hair count increases of 40% were observed in PSO-treated men at 24 weeks, whereas increases of 10% were observed in placebo-treated men (*P* < 0.001). Adverse effects were not different in the two groups.

## 1. Introduction

Androgenetic alopecia (AGA) is the most common cause of hair loss in men and affects up to 70% of men in later life and especially those aged over 50 years [1–3]. Genetic factors and androgens primarily underlie the pathogenesis of AGA. Hair follicles become gradually miniaturized and spend less time in the active phase (the anagen phase) and more time in the resting phase (the telogen phase) of hair growth [4]. Furthermore, it is known that dihydrotestosterone (DHT) is a major player in the process [5].

Topical minoxidil and oral finasteride have been approved by the FDA for the treatment of AGA, but only about 30% of patients persist with medication over a year in private practice [6–8]. Oral finasteride was found to decrease libido and ejaculate volume or cause erectile dysfunction, whereas topical minoxidil can cause scaling and itching of the scalp. Due to these adverse effects, patients seem to be drawn to alternative treatments with fewer side effects. In this context, many natural products have been tested as potential alternative therapies for hair loss. Some products, such as green tea and saw palmetto, have demonstrated therapeutic potential for the treatment of AGA and benign prostatic hyperplasia (BPH) via the inhibition of 5*α*-reductase activity [9, 10]. Pumpkin seed oil (PSO) has also been reported to be an effective treatment for symptomatic BPH [11]. Its actions have been suggested to be due to phytosterols, which are known to inhibit 5*α*-reductase and to have antiandrogenic effects in rats [12]. However the effects of PSO on AGA have not been established. We hypothesized that PSO is an effective, safe agent for the treatment of men with AGA, and thus we evaluated the efficacy and tolerability of PSO for treatment of hair growth in male patients with mild to moderate AGA.

## 2. Materials and Methods

### 2.1. Study Design

This study had a randomized, placebo-controlled, double-blind, controlled design. The study was approved by the Institutional Review Board at Pusan National University Yangsan Hospital and was performed in accordance with the principles of the Declaration of Helsinki. Written informed consent was obtained from all subjects, which were recruited by advertising, before enrollment. Eligible patients had mild to moderate hair loss classified as the Norwood-Hamilton type II, III, III Vertex, IV, and V [13]. Ninety adults between the ages of 20 and 65 years with mild to moderate AGA were initially enrolled at a tertiary hospital in Yangsan. The subjects had not applied any topical treatment or taken any medication or supplement for hair loss, including finasteride, any other 5*α*-reductase inhibitor, minoxidil, steroids, or hormonal products, during the 3 months prior to study commencement. For safety reasons, candidates with an aspartate aminotransferase (AST) or alanine aminotransferase (ALT) serum level greater than 60 mg/dL or a creatinine level greater than 1.5 mg/dL were excluded. Four participants met the exclusion criteria and ten participants declined to participate. Finally 76 (84.4%) participants were enrolled. After taking baseline measurements, participants were randomly assigned to one of two groups: the intervention group (*n* = 37), members of which received 400 mg of PSO (Octa Sabal Plus^®^) per day in the form of capsules, or the control group (*n* = 39), members of which received a placebo. Two capsules (100 mg per capsule) of PSO were taken by subjects in the intervention group 30 minutes before breakfast and dinner (total of 4 capsules per day) for a period of 24 weeks. Subjects in the control group were given the same quantity of placebos two times per day for 24 weeks. Capsules were supplied with visually identical forms by Dreamplus Co., Ltd. (Cheonan, Korea). Five participants in the intervention group and 7 in the control group dropped out during the study. The characteristics of these 12 participants were similar to those that completed the study. Subjects were assessed with respect to safety and compliance at every clinic visit (after 1, 4, and 12 weeks of treatment). Compliance was assessed by pill count. Reports of adverse events were collected throughout the study.

### 2.2. Randomization

Participants were assigned to the intervention and control groups using random numbers tables, and assigned identification numbers on recruitment. Randomization codes were held by Dreamplus Co., Ltd. The individual responsible for deciding on study eligibility and the individuals that conducted the measurements were unaware of the results of randomization.

### 2.3. Measurements

Body mass index was defined as weight (kg) divided by height squared (m^2^). A mercury sphygmomanometer was used to measure blood pressure (BP) in the sitting position after a 10-minute rest period. Two readings of systolic and diastolic BP were recorded at 3-minute intervals, and averages were included in the analysis.

Blood samples were taken at baseline and after 24 weeks of treatment after a 12 h fasting. Fasting blood sugar was measured using a glucose oxidase test method (LX-20, Beckman Coulter, Fullerton, CA, USA). Serum AST, ALT, *γ*-glutamyltransferase (GGT), and creatinine were determined using a Toshiba TBA200FR biochemical analyzer (Toshiba Co. Ltd., Tokyo). Serum-free testosterone was measured using a Coat-A-Count radioimmunoassay with gamma-10 (Shin Jin Medics, Korea).

### 2.4. Patient Self-Assessment

After 12 and 24 weeks of treatment, patient self-assessment of the efficacy of the treatment (self-rated improvement score) was based on a self-administered hair growth, using a Visual Analogue Scale (VAS), ranging from zero (representing the worst imaginable state) to 10 (the best imaginable state). Self-rated satisfaction with treatment was also measured using a 10-point VAS with 0 and 10 representing the lowest and highest satisfaction level, respectively.

### 2.5. Investigator Assessments

Pictures were taken of the vertex and superior frontal scalp of each patient at baseline and after 24 weeks of treatment using a standardized technique, as previously described [14]. A blinded investigator rated changes in scalp appearance relative to baseline (immediately prior to treatment commencement) in blinded fashion using a standardized 7-point rating scale as follows: greatly decreased (score of −3), moderately decreased (−2), slightly decreased (−1), unchanged (0), slightly increased (+1), moderately increased (+2), and greatly increased (+3) [15]. Investigator assessments were performed at 12 and 24 weeks.

### 2.6. Hair Analysis by Phototrichography

Hair changes including hair counts and diameters were assessed after 12 and 24 weeks of treatment versus baseline by phototrichography (Scalp & Hair Polarizing system, KC Technology, Seoul, Korea). Hair analysis using a phototrichography was performed by one technician. At baseline, the most severe site of baldness was recorded as target area of hair changes and the center of the phototrichogram probe was placed at this site. After 12 and 24 weeks of treatment, hair analysis was performed with confirmation of recorded target area. Hair counts were then recorded using a ×60 lens and the thickest hair diameter was recorded using a ×150 lens.

### 2.7. Statistical Analysis

The primary outcome variables were blinded investigator assessment and patient self-assessment scores. Secondary outcomes variables were changes in hair thickness and hair count. The calculated sample size was 35 patients per group for an 80% power to detect a difference in the mean investigator assessment score of 0.3, assuming a standard deviation of 0.5 in the primary outcome variables and an alpha error of 5%. When test data was unavailable, the last recorded data entry was entered in the analysis (the last observation carried forward method). Efficacy analysis was performed on an intention to treat (ITT) basis on participants that received at least one dose of PSO or placebo and that underwent at least one assessment postbaseline. The D'Agostino Pearson test was used to test for variable normality. Intergroup comparisons of baseline characteristics and of their changes after 24 weeks of treatment were performed using the two-sample Student's *t*-test for continuous variables or the chi-square test for categorical variables. Intragroup comparisons were performed using the paired Student's *t*-test. Repeated-measures ANOVA was used to evaluate intergroup differences in variables. *P* values of less than 0.05 were considered statistically significant. SPSS version 18.0 was used for the analysis.

## 3. Results

### 3.1. General Characteristics of the Study Subjects

Compliance was satisfactory and participants took more than 95% of the supplements in the intervention and control groups. Randomization was successful, as the two groups generated were comparable for most variables, and no significant differences were observed in baseline demographic, anthropometric, or clinical characteristics between the intervention and control groups (Tables 1 and 2). Furthermore, no statistically significant intergroup differences were observed for age at the onset of hair loss, family history of alopecia, or laboratory data at baseline. During the study period, the double-blind requirement was well maintained.

### 3.2. Patient Self-Assessment

No significant intergroup differences were observed for self-rated improvement (*P* = 0.514) or self-rated satisfaction scores (*P* = 0.214) for hair growth at 12 weeks. However, after 24 weeks, the self-rated improvement score in the intervention group was higher than in the control group (3.4 ± 2.9 in the intervention group versus 2.1 ± 2.0 in the control group, mean ± SD), and this difference was significantly different (*P* = 0.013 by the two sample Student's *t*-test). In addition, group self-rated satisfaction scores were also significantly different at 24 weeks (3.5 ± 2.9 in the intervention group versus 2.3 ± 2.0 in the control group, *P* = 0.003) (Table 3).

### 3.3. Investigator Assessment Using Photographs

**Figure 1:**
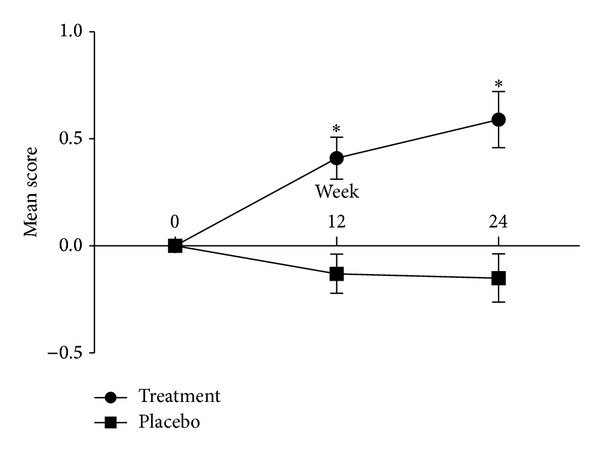
Investigator assessment of clinical response using a standardized 7-point rating scale during 24 weeks after study start. Data are expressed as mean with SE. *P < 0.001 by repeated-measures ANOVA. A standardized 7-point rating scale of hair growth compared with baseline (−3 = greatly decreased, −2 = moderately decreased, −1 = slightly decreased, 0 = no change, +1 = slightly increased, +2 = moderately increased, and +3 = greatly increased). Photographs of scalp hair were taken for hair counts and for assessments of hair growth by an expert panel.

**Figure 2:**
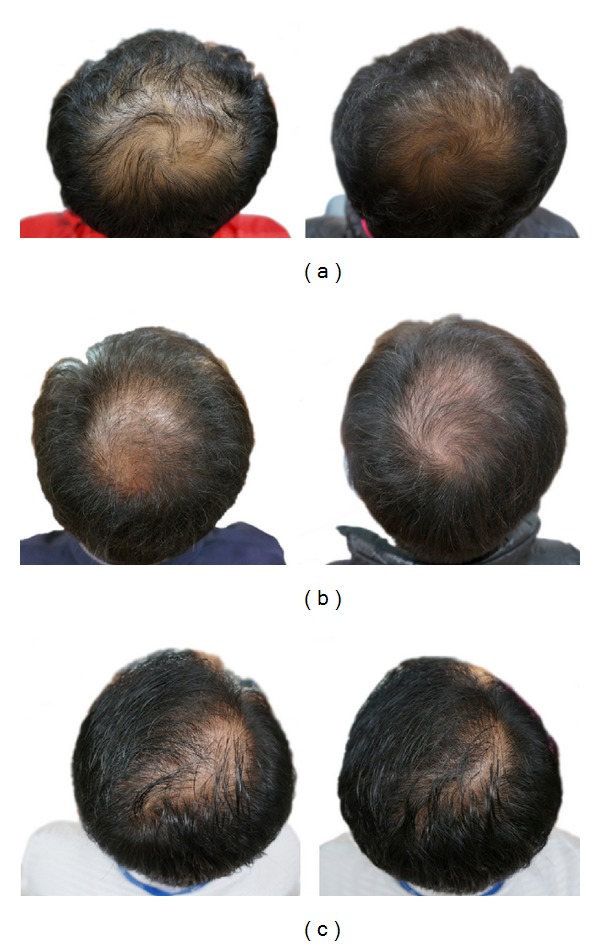
Representative photographs of patients at baseline and after 24 weeks of treatment with PSO.

Based on blinded investigator assessments, treatment with PSO was superior to treatment with placebo with respect to hair growth at 12 and 24 weeks (*P* < 0.001, all comparisons). In the control group, there was no initial improvement during the first 12 weeks, and then hair growth plateaued during the second 12 weeks (Figure 1). At 24 weeks, 2.7% (1/37) of subjects in the intervention group were assessed to have worsened by blinded investigator assessments; 51.4% (19/37) were rated as unchanged relative to baseline and 44.1% (17/37) were rated as slightly or moderately improved (Figure 2). On the other hand, at 24 weeks, 28.2% (11/39) of subjects in the control group were assessed to have worsened based on the investigator; 64.1% (25/39) were rated as unchanged relative to baseline and 7.7% (3/39) were rated as slightly or moderately improved. These results showed significant intergroup differences (data not shown, *P* = 0.002 by chi-square test).

### 3.4. Hair Analysis Using Phototrichography

**Figure 3:**
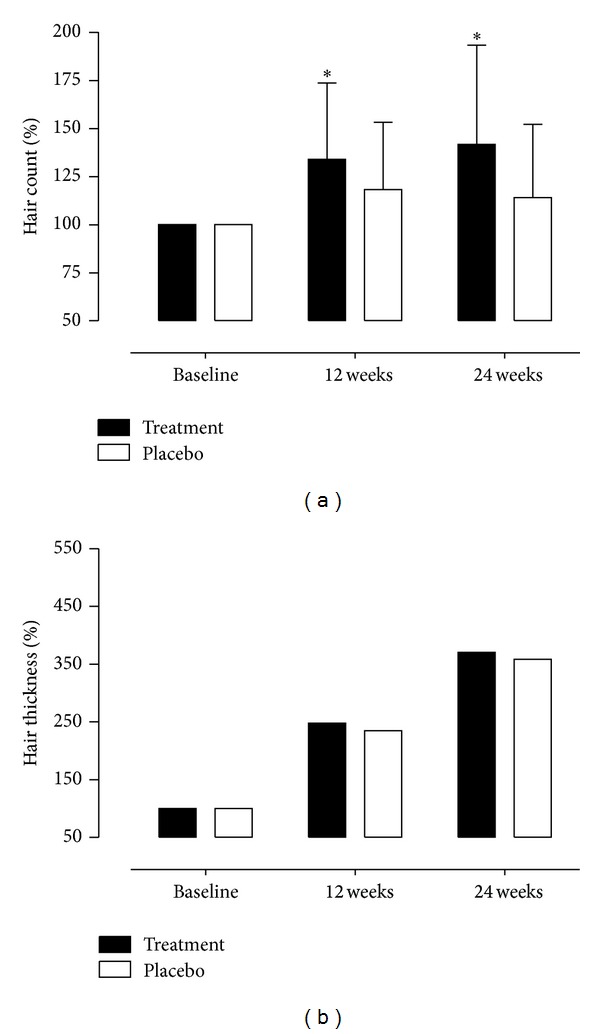
Changes in hair count (a) and thickness (b) during 24 weeks after study start. Data are expressed as mean with SD. Change (%) = (parameter at week 12 or 24-parameter at baseline)/(parameter at baseline) × 100. *P < 0.001 by repeated-measures ANOVA.

Hair changes, that is, hair counts and hair diameters, were measured by phototrichography at baseline and at 12 and 24 weeks. There were statistically significant differences in hair count changes during 24 weeks in the intervention group and the control group (Δ hair count per field of view using a ×60 lens; 6.2 ± 6.5 versus 1.8 ± 6.2; *P* = 0.004). However, changes in hair thickness during 24 weeks were similar in the groups (Δ hair thickness per field of view using a ×150 lens; 0.34 ± 0.03 versus 0.34 ± 0.02; *P* = 0.991). At 12 and 24 weeks, there were 30% and 40% mean increases in hair counts from baseline in PSO-treated men and 5% and 10% increases in hair count in placebo-treated men, which resulted in significant net increase of 25% and 30% (both, *P* < 0.001) at weeks 12 and 24, respectively, in the intervention group as compared with the placebo group (Figure 3).

### 3.5. Safety

Most of the subjects completed the protocol without adverse symptoms. One subject in the intervention group and one subject in the control group complained of a whole body itching sensation. One subject in the intervention group complained of mild abdominal discomfort. No changes in liver enzyme or creatinine were observed in the intervention group. Serum-free testosterone levels were unchanged in both groups (Table 3). No intergroup differences in blood pressure or glucose were observed during the study period (Table 4).

## 4. Discussion 

To our knowledge, this is the first randomized, double-blind, placebo-controlled trial to investigate the efficacy and tolerability of PSO in men with mild to moderate AGA. This study shows that PSO supplement during 24 weeks has a positive anabolic efect on hair growth and that this is due to the possible efects of 5-reductase inhibition in patients with mild to moderate male pattern hair loss.

AGA is the most common type of hair loss to affect both males and females after puberty. Although AGA is not a serious health problem, there is a strong demand for treatment and prevention due to the high level of interest people have in personal appearance in modern society. It is well known that finasteride and minoxidil are effective treatments for androgenetic alopecia and both have been approved by the FDA for this purpose, but some patients do not like taking medicine in the long term, because of possible side effects. For example, finasteride can decrease libido and ejaculate volume or cause erectile dysfunction, whereas minoxidil can cause scaling and itching of the scalp [6, 7].

Herbal therapies have been used to treat baldness since ancient times in the Ayurveda, Chinese, and Unani traditional medicinal systems [4]. Natural products, such as grape seed and rosemary oil, have been shown to be possible alternative treatments for AGA due to improved scalp blood flow [16, 17]. Several studies have reported that the polyphenols in green tea might be useful for treating AGA by inhibiting 5*α*-reductase activity [9, 10].* Cuscuta reflexa *exhibited hair growth promotion via 5*α*-reductase inhibitory effect and this herbal extract was highlighted as a potential treatment for hair loss [18]. Soymetide-4 was one of the herbal product-suppressing alopecia and ginseng (*Panax ginseng*) was also used for scalp treatment limiting hair loss with anti-inflammatory and blood circulation effect [19, 20].* Eclipta alba* extract and* Zizyphus jujuba* essential oil showed possibility of alternative treatment of alopecia [21, 22].

Saw palmetto (*Serenoa repens*) has been used as a natural treatment for androgenetic alopecia and has a similar mechanism [23]. Interestingly, in a recent study, it was found that 38% of males with AGA which received* Serenoa repens* at 320 mg every day for 24 months showed increased hair growth. The authors concluded that it could be used as an alternative treatment for mild to moderate AGA and that it is more effective than finasteride in this respect [24]. PSO is rich in beneficial nutrients, such as essential fatty acids, *β*-carotenes, lutein, *γ*- and *β*-tocopherols, and phytosterols [25]. It has been reported in several animal studies that PSO inhibits testosterone-induced hyperplasia of the prostate and suggested that PSO may be beneficial for the management of BPH [10, 24]. Another study confirmed that the intake of PSO at 320 mg/day over 12 months is clinically safe and effective as a complementary treatment for BPH [11]. Although the action mechanism is still unclear, previous animal studies have suggested that PSO may inhibit 5*α*-reductase, which produces DHT from testosterone [11, 12, 26].

Our study has some limitations, which include a lack of histological confirmation of the action mechanism of PSO. In addition, DHT and prostate specific antigen (PSA) levels were not measured for identification of mechanism of PSO; therefore we could not explain the exact action of PSO on AGA patients. However, the simplified International Index of Erectile Function (IIEF-5) was surveyed at baseline and 24 weeks because of identification of changes of libido and no significant intergroup differences were observed (data not shown, *P* = 0.774). The final limitation is degree of accuracy when assessing changes of hair by phototrichography, even though hair analysis was performed with confirmation of recorded target area in baseline. Nevertheless, our results have a value because current study was conducted based on the randomized controlled trials which evaluated hair changes using photographs as well as phototrichography.

Despite of these limitations, this double-blinded study did involve 76 subjects and, to the best of the authors' knowledge, is the first study to examine the long-term efficacy of PSO on AGA. The study shows that PSO could improve AGA and that it should be considered a potential alternative treatment. However, replication will be needed in order to confirm the results of this first-stage study and additional studies are required to elucidate the mechanism responsible for the positive effects of PSO on AGA.

## Figures and Tables

**Table 1:** Basal characteristics of subjects.

	Treatment (*N* = 37)	Placebo (*N* = 39)	*P* value*
Age (years)	45.6 ± 9.9	46.8 ± 10.3	0.615
Age of onset of hair loss (years)	34.2 ± 8.6	36.8 ± 9.8	0.223
The Norwood-Hamilton grade			0.373
Stage 2	8 (21.6)	4 (10.3)	
Stage 3	5 (13.5)	2 (5.1)	
Stage 3V	11 (29.7)	15 (38.5)	
Stage 4	7 (18.9)	8 (20.5)	
Stage 5	6 (16.2)	10 (25.6)	
Family history of androgenetic alopecia	27 (73.0)	27 (69.2)	0.719
Smoking (pack-year)	5.9 ± 8.8	2.6 ± 6.4	0.067
Body mass index (kg/m^2^)	24.7 ± 2.9	24.5 ± 2.9	0.734

Data are expressed as means ± SD or frequency (percent).

*Two-sample Student's *t*-test or chi-square test.

**Table 2:** Clinical and biochemical characteristics at the start of the study and after 24 weeks of treatment.

Variables	Treatment (*N* = 37)			Placebo (*N* = 39)			*P* value*	*P* value^†^
	Week 0^‡^	Week 24	Δ 24−0 weeks	Week 0^‡^	Week 24	Δ 24−0 weeks		
Body weight (kg)	72.9 ± 10.1	72.4 ± 10.2	−0.5 ± 1.6	71.1 ± 10.6	71.0 ± 10.8	0.1 ± 2.3	0.079, 0.747	0.419
WC (cm)	89.0 ± 7.3	85.8 ± 6.7	−3.3 ± 3.6	87.1 ± 6.1	83.7 ± 6.5	−3.4 ± 6.1	**0.000, 0.001**	0.890
Systolic BP (mmHg)	125.6 ± 15.5	124.4 ± 13.8	−1.2 ± 11.3	128.3 ± 14.6	129.3 ± 13.8	1.0 ± 15.8	0.518, 0.710	0.496
Diastolic BP (mmHg)	84.1 ± 12.8	84.0 ± 10.7	−0.1 ± 10.0	85.9 ± 13.6	85.2 ± 10.7	−0.8 ± 13.9	0.948, 0.731	0.813
Fasting glucose (mg/dL)	109.9 ± 30.0	112.9 ± 14.9	3.1 ± 27.2	104.4 ± 16.4	114.5 ± 22.4	10.1 ± 23.4	0.499, **0.011**	0.229
AST (IU/L)	22.0 ± 3.9	22.9 ± 4.6	0.9 ± 4.7	22.7 ± 4.1	25.7 ± 6.4	3.0 ± 5.8	0.246, **0.002**	0.091
ALT (IU/L)	20.3 ± 8.5	22.7 ± 11.6	2.3 ± 10.4	23.5 ± 8.7	27.2 ± 13.3	3.6 ± 11.1	0.183, **0.049**	0.603
GGT (IU/L)	33.0 ± 29.7	31.4 ± 17.6	−1.6 ± 18.5	38.5 ± 25.3	40.3 ± 27.4	1.8 ± 12.6	0.603, 0.378	0.351
Creatinine (mg/dL)	0.92 ± 0.13	0.91 ± 0.14	−0.01 ± 0.08	0.96 ± 0.13	0.96 ± 0.14	−0.00 ± 0.11	0.290, 0.827	0.658
Free testosterone (pg/mL)	8.5 ± 3.2	9.5 ± 3.1	0.9 ± 3.7	10.9 ± 3.6	10.2 ± 3.4	−0.7 ± 3.5	0.135, 0.235	0.055

AST: aspartate transaminase; ALT: alanine transaminase; BP: blood pressure; GGT: gamma-glutamyltransferase; WC: waist circumference.

Data are means ± SD. Δ 24-0 weeks and difference compared with baseline.

*Paired Student's *t*-test within group.

^†^Two-way repeated measures ANOVA over time between groups.

**Table 3:** Patients assessments of clinical response.

	Treatment (*N* = 37)		Placebo (*N* = 39)		*P* value*
	12 weeks	24 weeks	12 weeks	24 weeks	Weeks 12 and 24
Self-improvement^1^	2.9 ± 2.5	3.4 ± 2.7	2.5 ± 2.8	2.1 ± 2.0	0.514, **0.013**
Self-satisfaction^1^	3.4 ± 2.9	3.5 ± 2.9	2.6 ± 2.6	2.3 ± 2.0	0.214, **0.003**

Data are expressed as mean with SD.

^1^Patients provided ratings of improvement from 0 (no change or worse) to 10 (complete recovery) and satisfaction from 0 (complete disappointment) to 10 (full satisfaction) on Visual Analogue Scales.

*Two-sample Student's *t*-test between groups.

**Table 4:** Abnormal clinical findings at the start of the study and after 24 weeks of treatment.

	Treatment (*N* = 37)		Placebo (*N* = 39)		*P* value
	Week 0	Week 24	Week 0	Week 24	Weeks 0 and 24
SBP ≥ 140 or DBP ≥ 90 mmHg*	13 (35.1)	13 (33.3)	12 (32.4)	13 (33.3)	0.764, 0.933
Fasting glucose ≥ 126 mg/dL*	6 (16.2)	8 (21.6)	5 (12.8)	10 (25.6)	0.674, 0.680
AST or ALT ≥ 1.5 × upper limit of normal**	0 (0.0)	1 (2.7)	0 (0.0)	2 (5.1)	NA, 0.520
Creatinine ≥ 1.5 mg/dL**	0 (0.0)	1 (2.7)	0 (0.0)	0 (0.0)	NA, 0.487

AST: aspartate transaminase; ALT: alanine transaminase; DBP: diastolic blood pressure; SBP: systolic blood pressure.

Data are expressed as frequency (percent). *Chi-square test or **Fisher's exact test.
